# Identification and Analysis of Crucial Genes in *H*. *pylori*-Associated Gastric Cancer Using an Integrated Bioinformatics Approach

**DOI:** 10.1155/2023/8538240

**Published:** 2023-02-01

**Authors:** Wei Ding, Huaji Jiang, Nianyuan Ye, Ling Zhuang, Zhiping Yuan, Yulin Tan, Wenbo Xue, Xuezhong Xu

**Affiliations:** ^1^Department of General Surgery, Wujin Hospital Affiliated to Jiangsu University, Changzhou 213017, China; ^2^Department of General Surgery, The Wujin Clinical College of Xuzhou Medical University, Changzhou 213017, China; ^3^Changzhou Key Laboratory of Molecular Diagnostics and Precision Cancer Medicine, Changzhou 213017, China; ^4^Department of Gastroenterology, Wujin Hospital Affiliated with Jiangsu University, Changzhou 213017, China

## Abstract

**Background:**

The relationship between *H. pylori* infection and gastric cancer (GC) has been widely studied, and *H. pylori* is considered as the main factor. Utilizing bioinformatics analysis, this study examined gene signatures related to progressing *H. pylori*-associated GC.

**Materials and Methods:**

The dataset GSE13195 was chosen to search for abnormally expressed genes in *H. pylori*-associated GC and normal tissues. The TCGA-STAD database was chosen to verify the expression of key genes in GC and normal tissues.

**Results:**

In GSE13195, a total of 332 differential expression genes (DEGs) were screened. The results of weighted gene co-expression network analysis showed that the light cyan, plum2, black, and magenta4 modules were associated with stages (*T*3, *T*2, and *T*4), while the orangered4, salmon2, pink, and navajowhite2 modules were correlated with lymph node metastasis (*N*3, *N*2, and *N*0). Based on the results of DEGs and hub genes, a total of 7 key genes (ADAM28, FCER1G, MRPL14, SOSTDC1, TYROBP, C1QC, and *C*3) were screened out. These gene mRNA levels were able to distinguish between normal and *H. pylori*-associated GC tissue using receiver operating characteristic curves. After transcriptional level verification and survival analysis, ADAM28 and C1QC were excluded. An immune infiltration study revealed that key genes were involved in regulating the infiltration levels of cells associated with innate immune response, antigen presentation process, humoral immune response, or *T*cell-mediated immune response. In addition, drugs targeting FCER1G and TYROBP have been approved and are under investigation.

**Conclusion:**

Our study identified five key genes involved in *H. pylori*-associated GC tumorigenesis. Patients with higher levels of *C*3 expression had a poorer prognosis than those with lower levels. In addition, these key genes may serve as biomarkers and therapeutic targets for *H. pylori*-associated GC diagnosis, targeted therapy, and immunotherapy in the future.

## 1. Introduction

Incidence of gastric cancer (GC) is the sixth highest of all cancer types, with approximately 1,089,103 cases worldwide. GC is also the third leading cause of cancer death, with approximately 769,000 deaths each year [1]. The number of new cases of GC in China approaches 0.5 million each year [2]. Currently, the 5-year survival rate of GC patients is 32%, and more than 50% of patients are diagnosed with advanced cancer [3]. So far, surgery remains the only cure for GC [4]. The Human Genome Project is nearing completion and next-generation sequencing is being widely applied; researchers have made great progress in the study of the mechanism of GC occurrence and development [5]. The new medical model of the cross-development of sequencing technology and bioinformatics utilizes genomics and proteomics to guide targeted therapy, enabling GC patients to receive individualized and precise treatment [6]. To decrease the high incidence and mortality of GC, early detection and diagnosis are urgently needed, as well as new biomarkers for the disease. Although technology has advanced considerably, there is still an urgent need for efficient and timely diagnostic methods and new GC-specific biomarkers.

Various risk factors affect the incidence of GC, including *Helicobacter pylori* (*H. pylori*) infection, gender, poor dietary habits, and smoking [7]. Of these, *H. pylori* infection, which often leads to gastritis, followed by gastric atrophy and gastrointestinal metaplasia, is most closely related to GC [8]. Currently, the detection of *H. pylori* and its eradication therapy can reduce the risk of GC [9]. Mechanistically, the toxic effects of *H. pylori*-producedcytotoxicity-associated gene A (CagA) and vacuolar cytotoxicity A (VacA) proteins on gastric mucosal cells can trigger a series of complex biological effects, including release of proinflammatory cytokines, recruitment of immune cells, and stimulation of the survival of gastric epithelial cells [10, 11]. *H. pylori* inhibits phagocytic activity and *T* cell function during infection, while catalyzing the formation of urea to ensure its survival in harsh low pH conditions. Furthermore, *H. pylori* metabolism byproducts damage epithelial cells of the host and contribute to the carcinogenesis of *H. pylori* infection [12]. Despite numerous studies on *H. pylori*, it remains unclear whether *H. pylori* is only involved in the initiation of gastric tumor processes, or whether it affects the mechanisms of tumor progression.

In recent years, immunotherapy, as a novel treatment method, mainly induces antitumor effects by modulating the immune system and has made revolutionary progress in the treatment of gastric cancer [13]. The tumor microenvironment (TME) is a complex ecosystem consisting of immune cells coming in many forms and other acellular components of the extracellular matrix with marked heterogeneity. In the TME, tumor cells and immunomodulators interact dynamically to produce positive immunotherapy responses [14]. The immune microenvironment of GC itself is in a dynamic change, and whether the addition of *H. pylori* will make it more complicated.

In this article, based on the GSE13195 dataset and the TCGA-STAD dataset, we used a series of bioinformatics research methods to explore the dysregulated genes and mechanisms in *H. pylori*-associated GC tissues and to find possible biomarkers and targeted drugs.

## 2. Materials and Methods

### 2.1. Data Collection and Analysis

We selected the dataset GSE13195 from the Gene Expression Omnibus (GEO, https://www.ncbi.nlm.nih.gov/geo/) for our study [15]. The dataset was derived from GPL5175 (Affymetrix Human Exon 1.0 ST Array) and contained *H. pylori*-associated GC and normal tissues from 25 patients. The dataset also included patients' pathological information, tumor stages (*T*2, *T*3, and *T*4), and lymph node metastasis (*N*0, *N*2, and *N*3). Subsequently, Sangerbox Tools (https://www.sangerbox.com/) were used for normalized raw data as well as multiarray analysis (“lima” package) [16]. Finally, 134 downregulated genes and 198 upregulated genes were obtained according to the screening conditions of *P* value <0.05 and |logFC| > 1.

### 2.2. Functional and Pathway Enrichment Analysis

Genes differentially expressed were functionally enriched using DAVID v6.8 (Database of Annotations, Visualization, and Integrated Discovery, https://david.ncifcrf.gov/home.jsp) [17]. These include Gene Ontology (GO) enrichment analysis and Kyoto Encyclopedia of Genes and Genomes (KEGG) pathway analysis.

### 2.3. Gene Set Enrichment Analysis (GSEA)

To more accurately determine the functions of differential genes, we performed GSEA using Sangerbox Tools on the basis of normal tissues and *H. pylori*-associated GC tissues [16]. The reference gene set is c2.cp.kegg.v7.0.

### 2.4. Screen for Tumor Progression-Related Modules and Central Genes by Weighted Gene Co-Expression Network Analysis (WGCNA)

Gene co-expression networks in *H. pylori*-associated GC tissues were constructed using Sangerbox Tools [16]. First, based on Pearson correlation analysis, 25 samples were clustered to identify outliers. Then, we set the soft threshold to 5 to achieve a scale-free topology. Subsequently, using a dynamic tree-cut approach, the genes were classified into different modules based on gene expression correlations. The expression similarity of module eigen genes was further used to cluster similar modules with a height of 0.85. Module membership (MM) is the correlation of gene expression profiles with module characteristic genes, and genes with MM ≥ 0.8 are considered hub genes [18]. The protein interaction network was mapped using the String online website (https://string-db.org/).

### 2.5. Validation of Key Genes

Key genes were selected from abnormally expressed genes and hub genes. Receiver operating characteristic (ROC) curves were drawn to calculate specificity and sensitivity. In order to verify the accuracy and reliability of the screened key genes, the gene expression data of GC patients in the TCGA-STAD dataset (including 34 normal samples, 20 *H. pylori*-associated GC samples, 157 *H. pylori*-unassociated GC samples, and 153 other samples) were used for validation (including mRNA expression level and survival analysis) in UALCAN online website (https://ualcan.path.uab.edu/) [19].

### 2.6. Immune Infiltration Analysis

According to the calculation method of the immune microenvironment score of CIBERSORT, the immune microenvironment analysis of *H. pylori*-associated GC tissues and normal tissues was performed [20]. We calculated enrichment scores for each immune-related cell population using ssGSEA to examine the relationship between key genes and immune infiltration. In addition, Spearman correlations between each hub gene expression and immune enrichment scores were calculated and tested.

### 2.7. Target Drug

**Figure 1:**
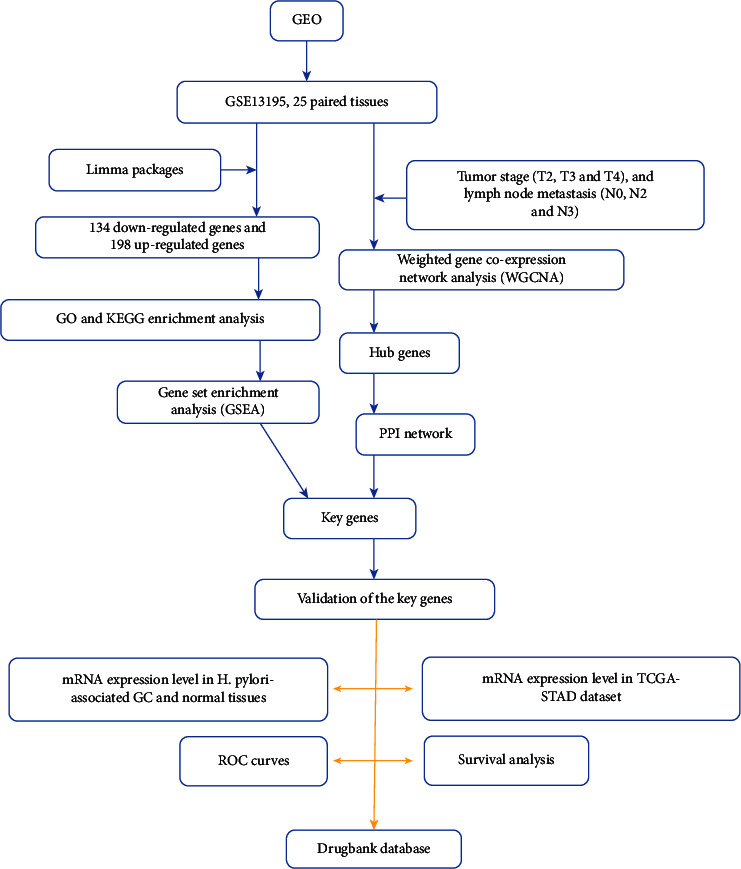
Flowchart of the study. GO: Gene Ontology; KEGG: Kyoto Encyclopedia of Genes and Genomes.

The DrugBank online analysis website (https://go.drugbank.com/) was used to find compounds that might act on key genes [21]. The flowchart of the study is provided in Figure 1.

## 3. Results

### 3.1. Data Collection and Acquisition of Differential Genes

**Figure 2:**
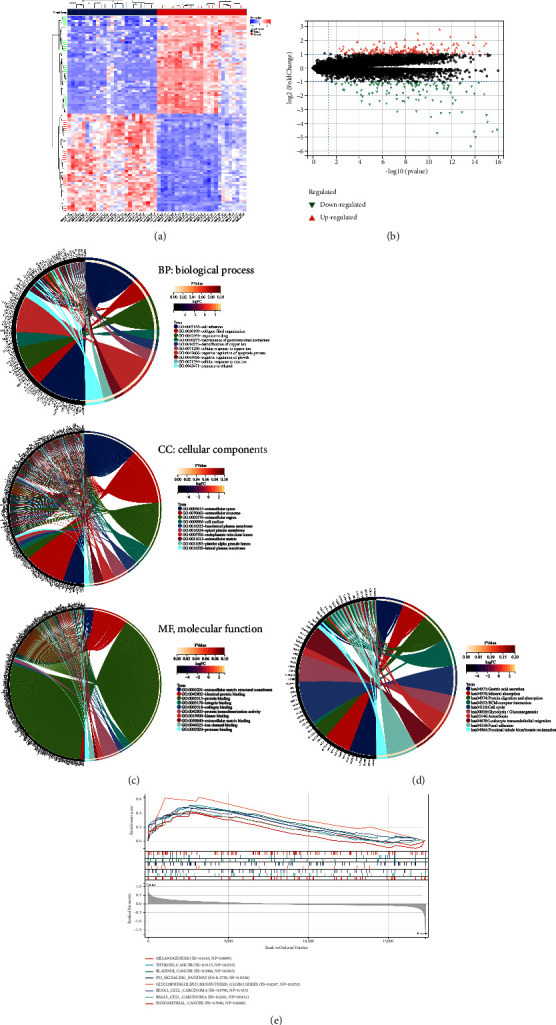
The analysis of differentially expressed genes. (a) Heatmap of differentially expressed genes. (b) Volcano map of differential expressed genes. (c) Gene ontology (GO) enrichment analysis (top 10). (d) Kyoto Encyclopedia of Genes and Genomes (KEGG) pathway enrichment analysis (top 10). (e) Gene set enrichment analysis.

The dataset GSE13195 from GEO was selected for this study. According to the screening conditions of *P* < 0.05 and |logFC| > 1, we found 332 differentially expressed genes (DEGs), including 198 that were upregulated and 134 that were downregulated (Figures 2(a) and 2(b)).

### 3.2. Functional and Pathway Enrichment Analysis

DAVID v6.8 was used for GO and KEGG enrichment analysis in order to better elucidate the functional and biological significance of the modules identified. GO biological process analysis showed that in terms of biological process, these differential genes were mainly related with cell adhesion, collagen fibril organization, response to drug, maintenance of gastrointestinal epithelium and detoxification of copper ion; in terms of cellular components, these differential genes were mainly located in extracellular space, extracellular exosome, extracellular region, cell surface, and basolateral plasm membrane; in terms of molecular functions, these differential genes mainly participated in extracellular matrix structural constituent, identical protein binding, protein binding, integrin binding, and collagen binding (Figure 2(c)). Furthermore, KEGG analysis revealed that these differential genes were highly involved in the regulation of gastric acid secretion, mineral absorption, protein digestion and absorption, ECM-receptor interaction, and cell cycle (Figure 2(d)).

### 3.3. Differential Gene Set Enrichment Analysis

GSEA was conducted to better elucidate how differential genes function. The eight KEGG pathways associated with DEGs are shown in Figure 2(e). They were melanogenesis, thyroid cancer, bladder cancer, P53 signaling pathway, glycosphingolipid biosynthesis, renal cell carcinoma, basal cell carcinoma, and endometrial cancer. Moreover, compared with normal tissues, these related pathways were hyperactivated in *H. pylori*-associated GC tissues.

### 3.4. Co-Expression Network Construction and Module Detection

**Figure 3:**
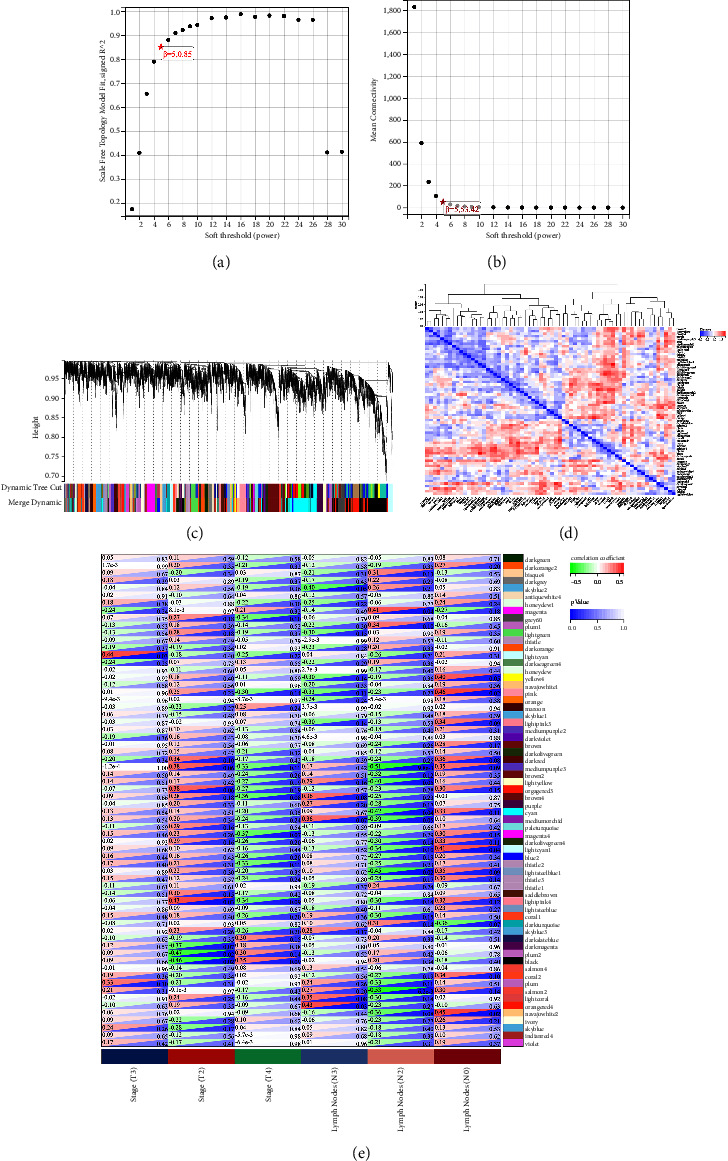
The weighted gene co-expression network analysis (WGCNA). (a) Analysis of the scale-free fit index for various soft-thresholding powers. (b) Analysis of the mean connectivity for various soft-thresholding powers. (c) Clustering dendrograms of GSE13195. (d) Module relationships. (e) Heat maps of the correlation between eigen gene and clinical traits of *H. pylori*-associated GC. GC: gastric cancer.

**Figure 4:**
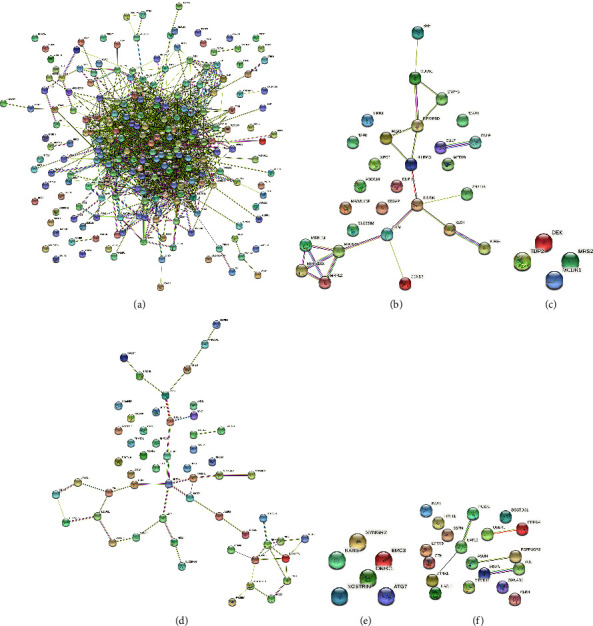
Protein-protein interaction (PPI) networks. (a) *T*3-related hub genes. (b) *T*2-related hub genes. (c) *T*4-related hub genes. (d) *N*3-related hub genes. (e) *N*2-related hub genes. (f) *N*0-related hub genes.

To find modules highly correlated with the progression of *H. pylori*-associated GC, samples of cancer tissues were used to construct a network of co-expression. We investigated the relationship between the scale-free topological fit index *R*^2^ and the soft threshold (power) in order to make the network scale-free. As shown in Figures 3(a) and 3(b), we chose a soft threshold (power) of 5 when *R*^2^ reached 0.85 for the first time. After the adjacency matrix was constructed, we transformed it into a topological overlap matrix. Genes were then sorted into different modules, performing a dynamic tree-cutting method. Different genes would be categorized into the same module if their expressions were significantly correlated. Finally, we got 66 modules; the module feature vector and clustering dendrogram are shown in Figures 3(c) and 3(d). Then, to identify modules that were highly correlated with the progression of *H. pylori*-associated GC, the correlation between tumor characteristics and each module was examined. As shown in Figure 3(e), among the 66 modules, modules light cyan, plum2, black, and magenta4 were most associated with stage (*T*3, *T*2, and *T*4) with *P* values below 0.05; modules orangered4, salmon2, pink, and navajowhite2 were associated with lymph node metastasis (*N*3, *N*2, and *N*0) were most correlated with *P* values below 0.05. We calculated MM and defined genes with MM ≥ 0.8 as central genes among the genes in selected modules and obtained a total of 318 hub genes. The protein interaction networks of these 318 hub genes in their respective categories are shown in Figure 4.

### 3.5. Acquisition and Specificity Analysis of Key Genes

**Figure 5:**
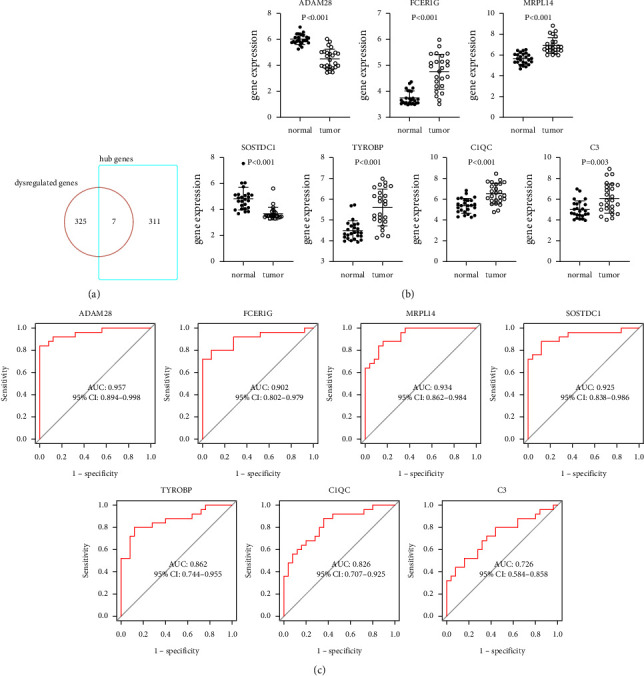
Validation of key genes in GSE13195. (a) Venn diagram. (b) mRNA expressions of key genes in GSE13195. (c) ROC curves of key genes in the diagnosis of *H. pylori*-associated GC. ROC: receiver operating characteristic. GC: gastric cancer.

Seven genes obtained by intersecting the differential genes and hub genes were defined as key genes, namely, ADAM28, FCER1G, MRPL14, SOSTDC1, TYROBP, C1QC, and *C*3 (Figure 5(a)). Their expression in the tissues of the GSE13195 dataset is shown in Figure 5(b). Among them, FCER1G, MRPL14, TYROBP, C1QC, and *C*3 were significantly highly expressed in *H. pylori*-associated GC tissues compared with normal tissues, while ADAM28 and SOSTDC1 were completely opposite. In addition, the ROC curves showed that the key genes were well predicted (AUC values: 0.957, 0.902, 0.934, 0.925, 0.862, 0.826, and 0.726, respectively) (Figure 5(c)). This suggested that seven key genes had the potential to be diagnostic markers for *H. pylori*-associated GC.

### 3.6. Validation and Survival Analysis of Key Genes

**Figure 6:**
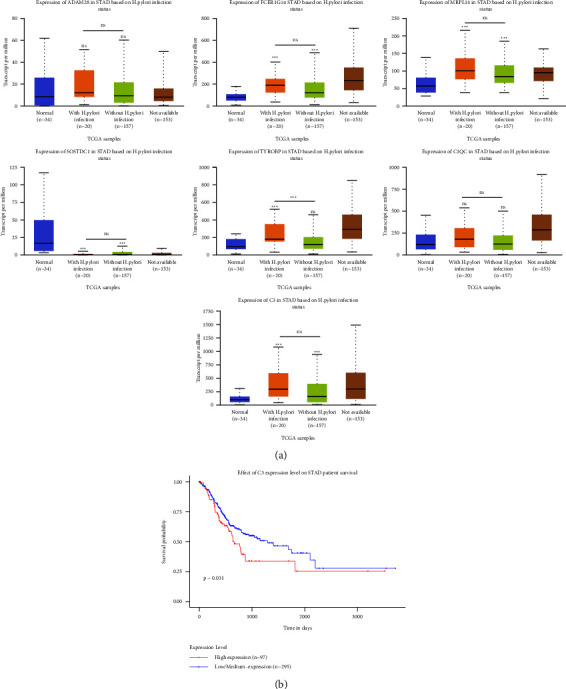
Validation of key genes in TCGA-STAD. (a) mRNA expressions of key genes in TCGA-STAD. (b) Association of *C*3 gene with overall survival of patients with GC.

Based on the TCGA database, boxplots of tumor samples and normal samples (including 34 normal samples, 20 *H. pylori*-associated GC samples, 157 *H. pylori*-unassociated GC samples, and 153 other samples) were generated for further validation of the key genes. As shown in Figure 6(a), the mRNA expression levels of the five key genes (FCER1G, MRPL14, *C*3, SOSTDC1, and TYROBP) were significantly different between tumor tissues and normal tissues, while ADAM28 and C1QC showed no significant differences. In addition, FCER1G, MRPL14, and *C*3 were abnormally high in *H. pylori*-associated and *H. pylori*-unassociated GC tissues compared to normal tissues; SOSTDC1 was abnormally low in *H. pylori*-associated and *H. pylori*-unassociated GC tissues. Interestingly, TYROBPHP was abnormally high in *H. pylori*-associated GC tissues compared to normal tissues but not in *H. pylori*-unassociated GC tissues. Furthermore, the expression of TYROBP was significantly increased in *H. pylori*-associated GC tissues relative to *H. pylori*-unassociated GC tissues. The expression levels of key genes were correlated with the prognosis of GC patients through survival analysis. According to the median expression value, GC patients were divided into a high expression group and low expression group. We found that patients with GC who expressed high levels of *C*3 had poorer overall survival, while the results of survival analysis of other genes were not statistically significant (Figures 6(b) and S1). Therefore, we removed ADAM28 and C1QC from the key genes.

### 3.7. Immune Infiltration Analysis

**Figure 7:**
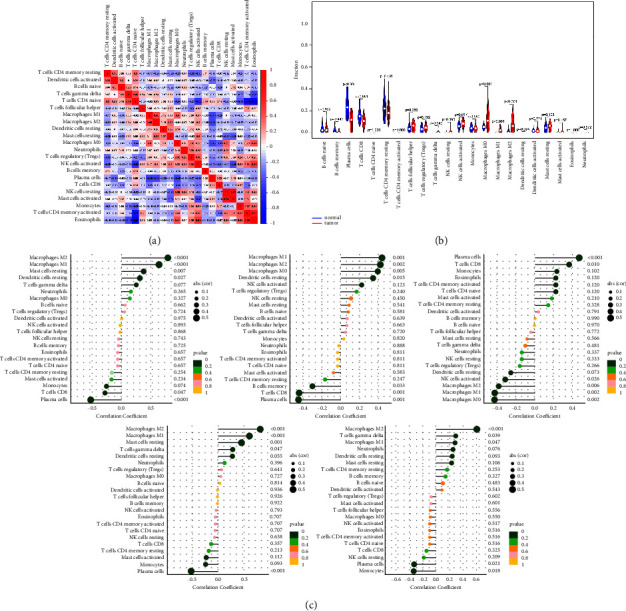
Immune infiltration analysis. The color means spearman correlation between the hub gene and the immune-related cell. (a) Correlation matrix of all 22 immune cells proportions. (b) Violin plot showed the proportions of 22 immune cells between normal tissues with *H. pylori*-associated GC tissues. (c) Influence of key genes on infiltration of 22 immune cells.

We performed immune microenvironment analysis on *H. pylori*-associated GC and normal tissues according to the CIBERSORT's calculation method of the immune microenvironment score. As shown in Figures 7(a) and 7(b), compared with normal tissues, *H. pylori*-associated GC tissues had stronger infiltration of activated NK cells, *M*0 macrophages, *M*1 macrophages, and *M*2 macrophages, but less infiltration of plasma cells and CD8 *T* cells, others are no different. We used ssGSEA to determine enrichment scores for immune-related cells. Spearman correlations between gene expression and immune enrichment scores for each hub were calculated and tested (Figure 7(c)). The results showed that FCER1G positively correlated with the infiltration of *M*2 macrophages, *M*1 macrophages, resting mast cells and resting dendritic cells, and negatively correlated with the infiltration of plasma cells and CD8 *T* cells. MRPL14 positively correlated with infiltration of *M*1 macrophages, *M*2 macrophages, *M*0 macrophages and resting dendritic cells, and negatively correlated with infiltration of plasma cells, CD8 *T* cells, and memory *B* cells. SOSTDC1 positively correlated with infiltration of plasma cells and CD8 *T* cells, and negatively correlated with infiltration of *M*0 macrophages, *M*1 macrophages, *M*2 macrophages, and activated NK cells. TYROBP was positively correlated with *M*2 macrophages, *M*1 macrophages, resting mast cells, and delta gamma *T* cell infiltration, and negatively correlated with plasma cell infiltration. *C*3 was positively correlated with infiltration of *M*2 macrophages, delta gamma *T* cells and *M*1 macrophages, and negatively correlated with infiltration of monocytes and plasma cells.

### 3.8. Possible Targeted Drugs

We used the DrugBank online website to search for possible targeted drugs in key genes. As shown in Table 1, for FCER1G, currently approved and under investigation drugs were benzylpenicilloyl polylysine and fostamatinib. Among them, benzylpenicilloyl polylysine acted as an agonist, while fostamatinib functioned as an inhibitor. For TYROBP, the currently approved and understudied drug was dasatinib, but it played a multitargeted role, and the specific mechanism remained to be further studied. The remaining compounds targeting key genes were poorly studied.

## 4. Discussion

Globally, GC is the third most common malignancy as well as the sixth most common cause of death [1]. The recent research showed that more than half of newly diagnosed patients were from developing countries (Eastern Europe, East Asia, and Central and South America) [22]. GC can occur due to a number of risk factors, including exposure to chemical carcinogens, environmental factors, genetic susceptibility, poor diet, and excessive alcohol intake [23]. However, infection with *H. pylori* remains the main cause of GC induction [24]. Despite the rapid development of targeted therapies and immunotherapies in recent years, there was still a lack of clinical effectiveness in treating some patients with GC [25]. It would be beneficial if more methods and targets could be found for treating GC. Based on transcriptome data analysis, our study identified DEGs associated with the occurrence and progression of *H. pylori*-associated GC, and provided some potential targets for the treatment of *H. pylori*-associated GC. Based on the GSE13195 and TCGA-STAD datasets, we identified five key genes, FCER1G, MRPL14, SOSTDC1, TYROBP, and C3, which presented different expression patterns in *H. pylori*-associated GC and normal tissues, where *C*3 may affect the prognosis of GC patients.

FCER1G is located on chromosome 1q23.3 and encodes the gamma subunit of the crystalline (Fc) region (Fc *R*) of an immunoglobulin fragment involved in various immune responses such as phagocytosis and cytokine release [26, 27]. Cellular effector functions are activated by the interaction between the Fc of immunoglobulins and the Fc *R* of immune cells, which in turn trigger destructive inflammation, immune cell activation, phagocytosis, oxidative burst, and cytokine release [28, 29]. FCER1G was implicated in the progression of several cancers, such as squamous cell carcinoma, multiple myeloma, and clear cell renal cell carcinoma [27, 29, 30]. In renal cancer, the high expression of FCER1G may be a functional basis for the induction of *M*2 macrophages by the increased secretion of IL-4. In addition, *M*2 macrophages can acquire their tumor suppressor function in part by suppressing cytotoxic *T* cells. This may explain the relevance of FCER1G to macrophage and *T* cell function [31]. These findings were consistent with our results that high expression of FCER1G was positively correlated with infiltration of *M*2 macrophages and negatively correlated with CD8 *T* cells.

MRPL14 is a highly conserved protein. One protein-binding site and two RNA-binding sites are located in the *C*-terminal region of MRPL14, which consists of a five-stranded beta barrel and two small alpha helices [32]. MRPL14 was found to be closely related to mitochondrial metabolism [33]. The conserved interaction of C7orf30 with MRPL14 promoted biogenesis of the mitochondrial large ribosomal subunit and mitochondrial translation [32]. However, research on the role of MRPL14 in cancers is currently still blank.

SOSTDC1 is a secreted protein with a glycosylated *N*-terminus that contains a *C*-terminal cysteine knot domain [34]. SOSTDC1 negatively regulates BMP (bone morphogenetic protein) signaling during cell proliferation, differentiation, and apoptosis, and also regulates various processes in development and cancer by regulating the Wnt pathway [35, 36]. Researchers have found that a lack of SOSTDC1 in GC patients was associated with a shorter survival rate. In gastric cancer, SOSTDC1 acts like a tumor suppressor, and its silencing can promote tumor growth and lung metastasis. SOSTDC1 significantly inhibits the SMAD-dependent BMP pathway, c-Jun activation, and transcription of c-Jun downstream targets [37]. In addition, SOSTDC1 regulates NK cell maturation and Ly49 receptor expression from nonhematopoietic and hematopoietic sources in a cellular-exogenous manner [38]. This seems to be contrary to the results we obtained in *H. pylori*-associated GC tissues, which needs to be further explored in the follow-up studies.

TYROBP, also known as DAP12, can noncovalently bind to activating receptors on the surface of various immune cells and mediate signal transduction and cell activation [39, 40]. There was evidence that patients with GC who overexpressed TYROBP had a poorer survival rate. Furthermore, TYROBP can stimulate macrophage activation, regulate tumor necrosis factor production, and induce tolerance [41]. TYROBP is involved in the interaction between tumor cells and macrophage *M*2 to enhance TGF-*β* secretion *in vitro* [42]. Our research partially confirmed this, but this part of the results still needs to be verified with large samples later.

Complement is an important part of the innate immune system. Previously, it was thought to be a network of proteins that released inflammatory mediators in response to microbial invasion [43]. A growing number of studies have shown that complement activation in the tumor microenvironment can delay local *T*-cell immunosuppression and chronic inflammation, thereby promoting tumor-promoting effects, ultimately promoting tumor immune escape, growth, and distant metastasis [44, 45]. *C*3 and downstream signaling molecules are involved in multiple biological processes of tumor cells, including tumor cell anchoring, proliferation, tumor-associated angiogenesis, matrix remodeling, migration, and invasion [46–48]. In GC, monocytes, TAMs, *M*2 macrophages, DCs, Tregs, and *T* cell exhaustion were significantly associated with *C*3 expression. An immunotherapeutic approach based on *C*3 could provide a potential biological target for GC [49].

Although we identified and confirmed 5 key genes that were highly correlated with the progression of *H. pylori*-associated GC, we were unable to perform multifaceted validation due to the small sample size of GSE13195 and the lack of studies of the same type. In addition, we did not perform experimental tests on key genes. It is critical to conduct larger sample studies as well as multicenter clinical trials to gain a deeper understanding of how genes are involved in *H. pylori*-associated gastric cancer.

## 5. Conclusion

In conclusion, we identified five key genes, FCER1G, MRPL14, SOSTDC1, TYROBP, and *C*3, associated with the occurrence of GC in *H. pylori* infection. Among them, *H. pylori*-associated GC patients with higher *C*3 expression had worse prognosis than those with lower expression. In addition, in the future, *H. pylori*-associated GC may be diagnosed and treated precisely by biomarkers and therapeutic targets related to these key genes.

## Figures and Tables

**Table 1:** Potential targeted drugs of key genes.

Genes	Potential drugs	Drug group	Actions
FCER1G	Benzylpenicilloyl polylysine	Approved	Agonist
	Fostamatinib	Approved, investigational	Inhibitor

TYROBP	Dasatinib	Approved, investigational	Multitarget

## Data Availability

The datasets used and/or analyzed during the current study are available from the corresponding author upon reasonable request.

## Supplementary Materials

Supplementary Materials Figure S1. Association of ADAM28, FCER1G, MROL14, SOSTDC1, TYROBP, and C1QC genes with overall survival of patients with GC.
