# Supplementary material for: Identification and Analysis of Crucial Genes in H. pylori-Associated Gastric Cancer Using an Integrated Bioinformatics Approach
Source: J Oncol. 2023 Feb 1;2023:8538240. doi: 10.1155/2023/8538240 (PMC9908346; doi:10.1155/2023/8538240)
Supplement: Supplementary Materials — Figure S1. Association of ADAM28, FCER1G, MROL14, SOSTDC1, TYROBP, and C1QC genes with overall survival of patients with GC. [file 8538240.f1.docx]

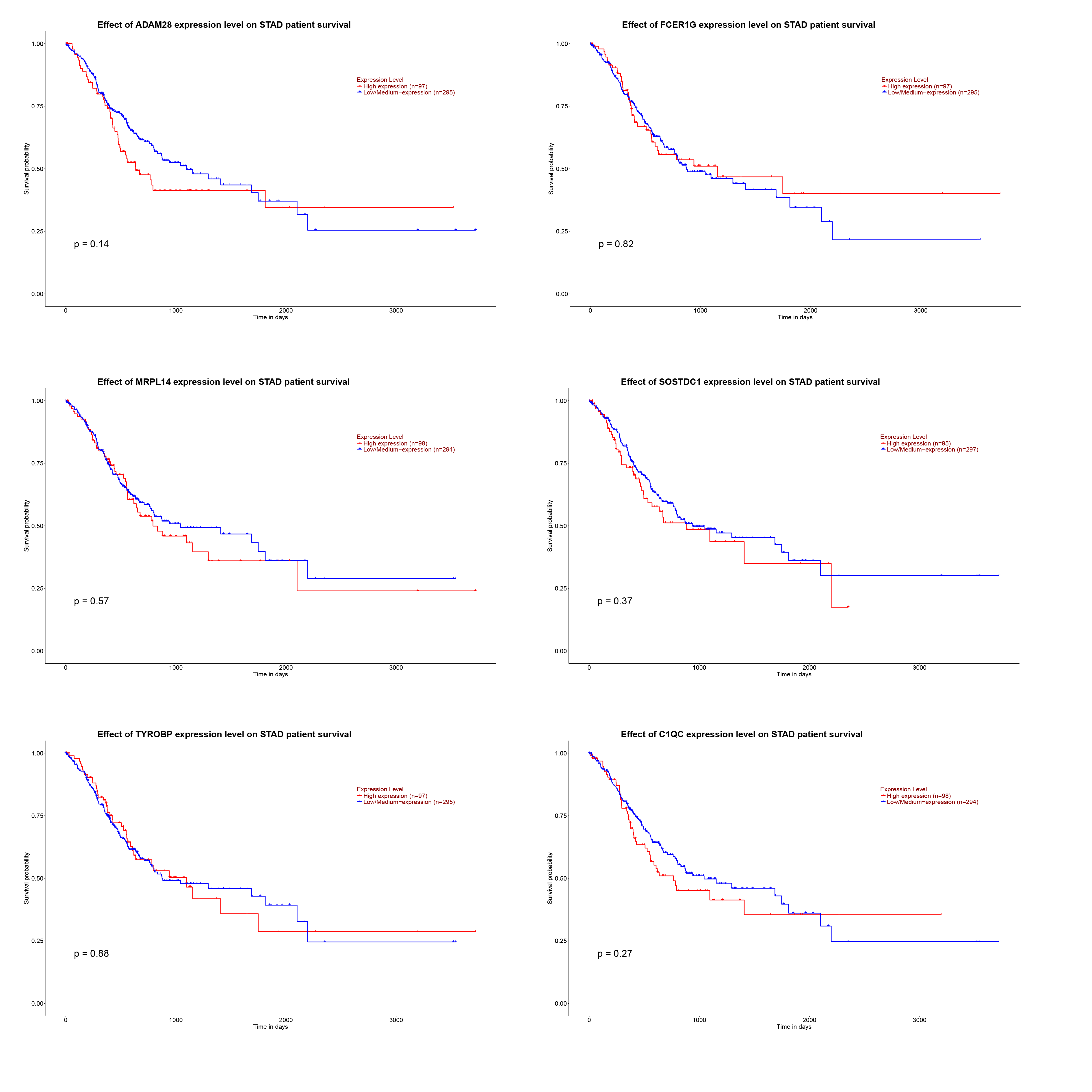


**Figure S1** Association of ADAM28, FCER1G, MROL14, SOSTDC1, TYROBP and C1QC genes with overall survival of patients with GC.
